# DNA-translocation-independent role of INO80 remodeler in DNA damage repairs

**DOI:** 10.1016/j.jbc.2023.105245

**Published:** 2023-09-09

**Authors:** Vladyslava Sokolova, Gahyun Lee, Amber Mullins, Preesha Mody, Shinya Watanabe, Dongyan Tan

**Affiliations:** 1 Department of Pharmacological Sciences, Stony Brook University, Stony Brook, New York, USA; 2 Program in Molecular Medicine, University of Massachusetts Chan Medical School, Worcester, Massachusetts, USA

**Keywords:** nucleosome, chromatin remodeling, DNA damage response, DNA repairs, DNA endonuclease

## Abstract

Chromatin remodelers utilize ATP hydrolysis to reposition histone octamers on DNA, facilitating transcription by promoting histone displacements. Although their actions on chromatin with damaged DNA are assumed to be similar, the precise mechanisms by which they modulate damaged nucleosomes and their specific roles in DNA damage response (DDR) remain unclear. INO80-C, a versatile chromatin remodeler, plays a crucial role in the efficient repair of various types of damage. In this study, we have demonstrated that both abasic sites and UV-irradiation damage abolish the DNA translocation activity of INO80-C. Additionally, we have identified compromised ATP hydrolysis within the Ino80 catalytic subunit as the primary cause of the inhibition of DNA translocation, while its binding to damaged nucleosomes remains unaffected. Moreover, we have uncovered a novel function of INO80-C that operates independently of its DNA translocation activity, namely, its facilitation of apurinic/apyrimidinic (AP) site cleavage by the AP-endonuclease 1 (APE1). Our findings provide valuable insights into the role of the INO80-C chromatin remodeler in DDR, thereby advancing our understanding of chromatin remodeling during DNA damage repairs.

Chromatin plays a crucial role in all DNA-based processes in eukaryotes. Nucleosomes, the primary structural units of chromatin, consist of 147 base pairs (bp) of DNA wrapped around a core of four histone proteins (H2A, H2B, H3, and H4) (1). DNA packaged into chromatin is continuously exposed to threats from exogenous and endogenous factors. However, the disk-like structure of nucleosome (2) and the intricate organization of chromatin present considerable challenges for efficient DNA damage repairs (3). Consequently, there is a need for chromatin structural rearrangements during the repair of damaged DNA *in vivo*.

The ATP-dependent chromatin remodelers are molecular machines that use the energy of adenosine triphosphate (ATP) hydrolysis to facilitate chromatin rearrangement in various nucleus processes. These remodelers feature a Superfamily-2 (SF2)-type ATPase motor that translocates DNA. Most remodelers also possess additional remodeler-specific subunits that confer their specific functions, enabling a range of remodeling reactions (4). However, the precise mechanisms by which these molecular machines employ their ATP-dependent chromatin remodeling activities on damaged chromatin remain poorly understood. Previous studies have indicated that the SWI/SNF chromatin remodeler enhances DNA accessibility *in vitro*, likely through its ATP-dependent DNA translocation property (5). Conversely, several studies have shown that single-stranded gaps or DNA nicks can block *in vitro* nucleosome repositioning by different remodelers (6, 7, 8, 9, 10). These studies suggest that DNA lesions, such as those caused by UV damage and chemotherapy, can disrupt remodeler activities at DNA damage sites.

INO80 is a versatile chromatin remodeler first identified in *Saccharomyces cerevisiae* from mutants defective in inositol/choline response (11). It modulates transcription, facilitates DNA repair, and maintains genome stability (12). Regarding its role in DNA damage response (DDR), early yeast studies indicate that mutations or deletion of the catalytic Ino80 subunit render the cells hypersensitive to agents that cause DNA damage (12, 13). Deletions of either of the two actin-related proteins (APRs) in INO80-C (denote the INO80 complex in yeast), Arp8 and Arp5, also give rise to DNA-damage-sensitive phenotypes indistinguishable from the *ino80*-null mutant (13). Further studies found that both Ino80 and Arp5 are enriched at UV-damaged DNAs in cells (14) and that loss of the Ino80 subunit leads to defective recruitments of Nucleotide Excision Repair (NER) factors to lesion sites (14). Additionally, the INO80-C complex has been directly implicated in repairing Double-strand break (DSB) in yeast through interactions with *γ*-H2AX (15). Despite these functional studies, the precise function of INO80 in different repair pathways and how INO80 interacts with repair factors to influence efficient repairs remain elusive.

To gain insights into the mechanism-of-action of the INO80 remodeler in damage repairs, we embarked on characterizing the interactions of INO80-C with damaged nucleosomes containing two distinct types of DNA lesions: AP sites and the *cis*-syn cyclobutene pyrimidine dimers (CPDs). AP sites, the most common lesions in genomic DNA, arise from spontaneous base loss and DNA glycosylase-catalyzed base release. They are repaired *via* the base excision repair (BER) pathway (16). On the other hand, CPDs are photo-damaged DNA products induced by UV irradiation and are commonly repaired through NER (17). CPD comprises 70 to 80% of the total photoproducts in cells (18). Deficiencies in the repair of DNA photoproducts like CPD lead to a hereditary disease called xeroderma pigmentosum (19). Here, we demonstrated that both types of DNA lesions inhibit the INO80-C-mediated DNA translocation. Our data also indicates that while DNA lesions do not diminish INO80-C binding to its substrates, they do impair ATP hydrolysis of the catalytic Ino80 subunit. Furthermore, we provide biochemical evidence that INO80-C stimulates the human APE1 endonuclease activity in an ATP-independent manner, suggesting a novel role of INO80-C remodeler in DDR independent of DNA translocation.

## Results

### Recombinant INO80-C complexes

**Figure 1:**
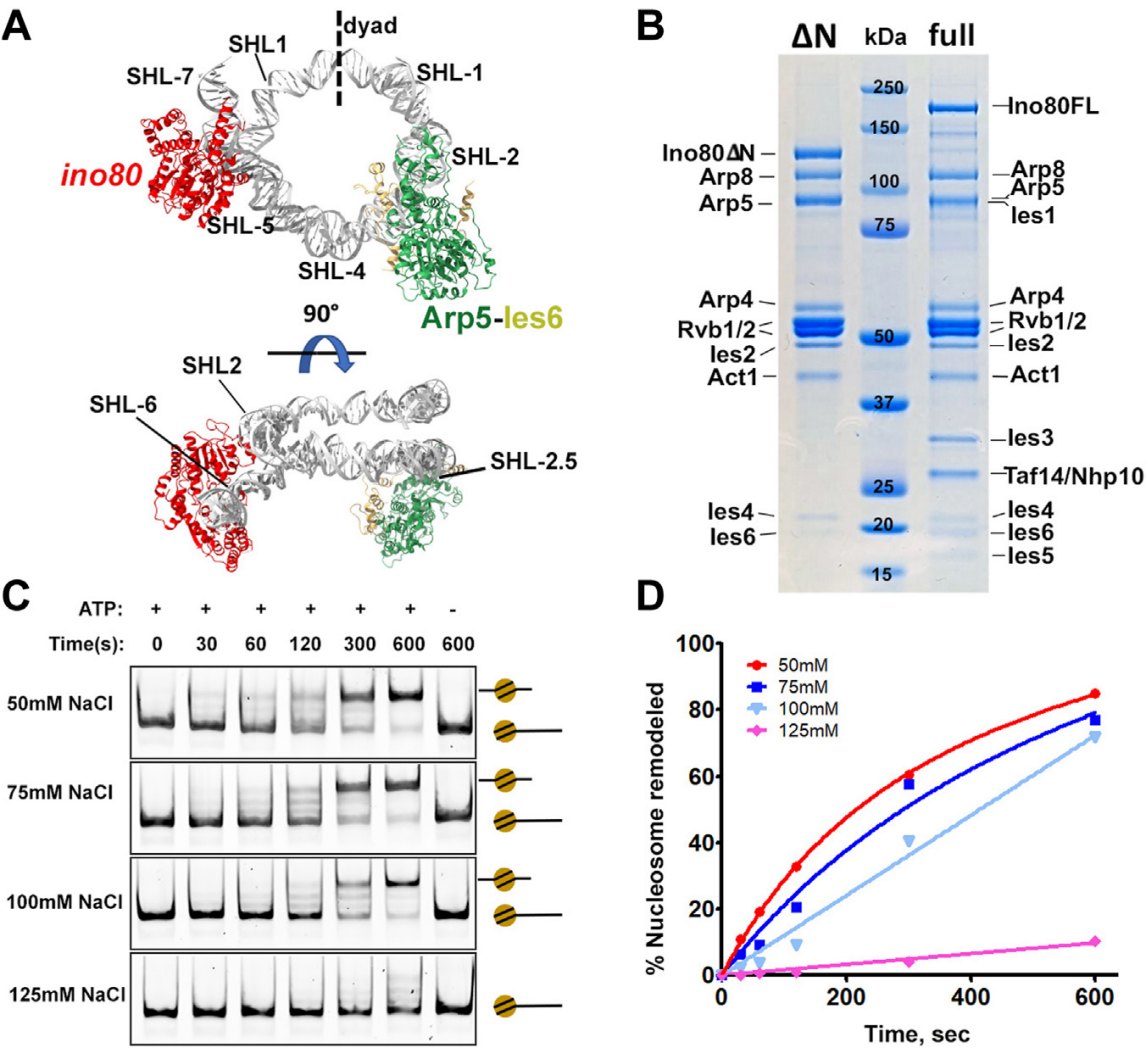
**Recombinant INO80-C complexes and the effect of monovalent cation in nucleosome sliding.***A*, structural model (PDB 6FML) in two different views showing the two INO80-C-engagement sites on nucleosomes. *B*, SDS-PAGE gel showing the purified recombinant INO80-C ΔN and the INO80-C (full) complex, with all subunits labeled on the side. *C*, INO80-C ΔN-dependent nucleosome sliding assay over 10 min in the presence of different concentrations of NaCl, shown on Native-PAGE. The positions of the bands for end-positioned and center-positioned nucleosomes are denoted by a schematic on the right side of the gels. *D*, quantification of (*C*), showing the percentage of remodeled nucleosomes over 10 min at different NaCl concentrations. Each data point represents one experiment.

The purification of the INO80 complex in both yeast and humans has revealed a highly conserved core structure (20, 21). In the current study, we generated the recombinant *S. cerevisiae* INO80 holo-complex (referred to as INO80-C hereafter) and a sub-complex (the INO80-C **Δ**N complex) (Fig. 1*B*). The latter mirrors the human INO80 core complex that is fully functional in all *in-vitro* remodeling assays (22). Beyond the central Ino80 subunit, the INO80-C **Δ**N complex comprises nuclear actin, three ARPs (Arp4, Apr5, and Apr8), Ies2, Ies4, Ies6, and two subunits sharing homology with the bacterial RuvB helicase (RvbL1 and RvbL2 in yeast, Tip49a and Tip49b in humans). Diverging from the norm observed in most chromatin remodelers that engage with nucleosomes at the superhelical-location (SHL) 2, INO80 uniquely cradles the nucleosome at SHL-6/6 and SHL-2/2. This dual interaction is facilitated by its catalytic subunit Ino80 and the Arp5-Ies6 module, respectively (23, 24) (Fig. 1*A*).

**Supporting Figure S1:**
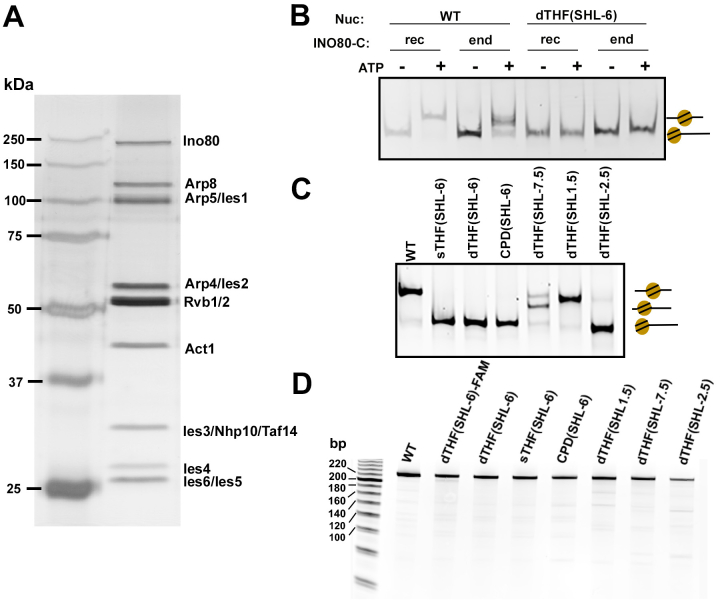
**Purification and activity of endogenous and recombinant INO80-C complexes.***A*, 15% Acrylamide SDS-PAGE gel showing subunits of the endogenous INO80-C complex. *B*, Nucleosome sliding assay shown on Native-PAGE. 140 nM end-positioned canonical (WT) or dTHF(SHL-6) mono-nucleosomes were mixed with 50 nM of either recombinant (rec) or endogenous (end) INO80-C complex. Reactions were initiated by adding 1 mM ATP and stopped after 20 min. *C*, Native-PAGE showing impeded sliding of the INO80-C ΔN complex on various damaged nucleosomes. 140 nM nucleosomes and 50 nM INO80-C ΔN complex were used in each reaction. Reactions were initiated by adding 1 mM ATP and stopped after 5 h. *D*, Denaturing gel of all the 601 Widom DNA fragments used for nucleosome reconstitution in the current study, showing negligible amounts of nicks/gaps present in the damaged DNAs.

To validate the nucleosome sliding capability of both INO80-C **Δ**N and INO80-C complexes, we employed a nucleosome sliding assay based on the electromobility shift assay (EMSA). Our results unequivocally confirm that both complexes efficiently reposition the nucleosome DNA from its end position to a center position (Figs. 1*C* and S1*B*). The outcome aligns with prior findings from both yeast and humans (22, 25).

Conventionally, nucleosome sliding facilitated by remodelers is conducted at room temperature under moderate concentration of mono- and divalent cations (50 mM in most published studies) (26). Our study supports this approach, substantiating the impact of increasing mono-valent salt levels on INO80-C **Δ**N-stimulated nucleosome sliding. Notably, at 75 mM NaCl, 77% of nucleosomes underwent remodeling within 10 min, in contrast to the 84% observed in control lacking NaCl supplementation (Fig. 1, *C* and *D*). With a rise to 100 mM NaCl, a marginal reduction in sliding activity was observed, resulting in 72% nucleosome remodeling. However, the introduction of 125 mM NaCl yielded an eight-fold decrease in sliding efficiency.

### Impact of DNA lesions on INO80-C-dependent nucleosome sliding

**Figure 2:**
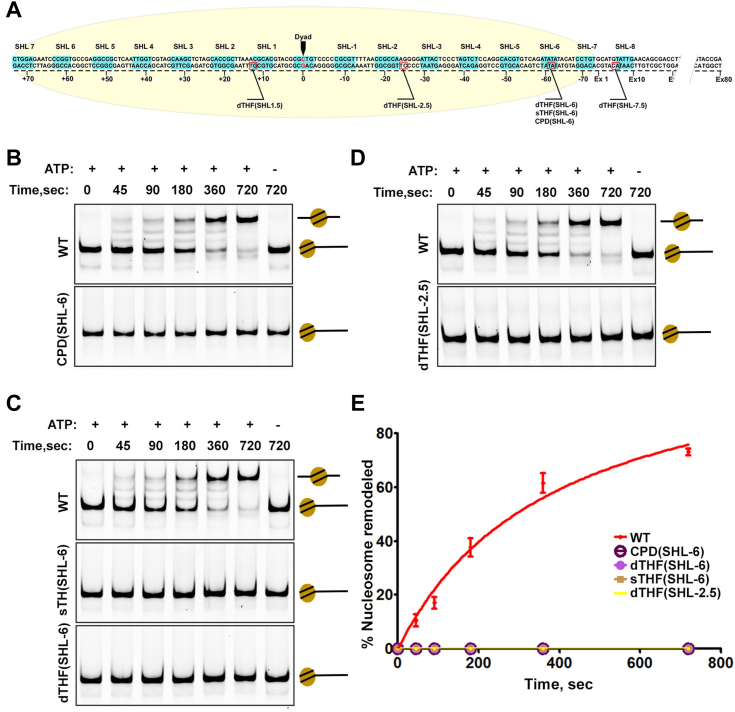
**DNA lesions on INO80-C binding sites inhibit its nucleosome sliding activity.***A*, schematic showing the locations (*red squares*) where THF and CPD were installed at the 601 DNA. *B*, representative native-PAGE showing normal nucleosome-sliding by INO80-C on canonical nucleosomes (WT), and impeded sliding on nucleosomes with CPD at the SHL-6 site. *C*, representative Native-PAGE showing nucleosome-sliding by INO80-C ΔN on canonical nucleosomes (WT), nucleosomes with single THF (sTHF), and nucleosomes with double TFH (dTHF) at SHL-6. *D*, representative Native-PAGE showing nucleosome-sliding by INO80-C ΔN on canonical nucleosomes and nucleosomes with dTHF at SHL-2.5. *E*, quantification of (*B*–*D*). Data are mean ± SD, n = 3.

Multiple studies have shown that DNA gaps at the SHL-5/-6 and SHL-2/-3 positions severely impede DNA translocation by INO80 (6, 27, 28). Notably, studies indicate that while gaps in proximity to the Arp5-Ies6 binding site (SHL-2/-3) disrupt remodeling, nicks at the exact location exert mild or negligible effects on INO80-driven DNA translocation (6, 28). To explore whether the aforementioned DNA lesions elicit analogous effects on INO80-C activities, we engineered end-positioned nucleosomes containing lesions at diverse SHL positions (Fig. 2*A*). Three types of lesions were employed (1): single tetrahydrofuran (sTHF) that emulates solvent-exposed AP sites (2), double THF (dTHF), and (3) CPD. Initially, these lesions were introduced at the SHL-6 position on the outer strand of the 0N80 601 Widom DNA fragment, followed by careful assessment of lesion incorporation using denaturing gels to ensure the absence of nicks and gaps (Fig. S1*D*). The outcomes notably revealed that all three lesions at the principal nucleosome contact site SHL-6 completely abolished DNA translocation by INO80-C ΔN (Fig. 2, *B*, *C* and *E*). Furthermore, the inhibition could not be surmounted through extended reaction periods, as evidenced by the absence of nucleosome sliding activity even after 5 h (Fig. S1*C*). Subsequently, we delved into the Arp5-Ies6 module binding site using nucleosomes harboring a dTHF lesion at SHL-2.5. This module is proposed to serve as a counter-grip, coupling ATP hydrolysis to nucleosome sliding, while the main Ino80 motor drives DNA translocation (24). As anticipated, the outcome revealed a complete halt in DNA translocation by INO80-C ΔN with the dTHF(SHL-2.5) nucleosome (Fig. 2, *D* and *E*), emphasizing the pivotal role of the Apr5-Ies6 module in INO80-driven DNA translocation.

The function of the Nhp10-Ies1-Ies3-Ies5 module, present in INO80-C but absent in INO80-C ΔN, remains enigmatic. A prior study revealed that the Nhp10 subunit contributes to INO80-C’s functions in DNA DSB repair (15), although it is dispensable for nucleosome sliding *in vitro*. We investigated whether this non-essential module endows INO80-C with the capability to translocate on damaged chromatin—an ability hindered in INO80-C ΔN. Our findings using both recombinant and endogenous INO80-C complexes indicated a complete inhibition of DNA translocation on dTHF(SHL-6) nucleosomes, mirroring the INO80-C ΔN complex results (Fig. S1*B*). This observation indicates that the Nhp10-Ies1-Ies3-Ies5 module does not confer the INO80-C complex with the capacity to circumvent DNA lesions during DNA translocation. We then assessed whether a single AP site alone could impede INO80-mediated nucleosome sliding. The results reiterated a complete loss of DNA translocation activity by the INO80-C ΔN complex on sTHF(SHL-6) nucleosomes (Fig. 2, *C* and *E*). These findings underscore the pivotal role of DNA integrity in ATP-dependent chromatin remodeling, establishing the heightened sensitivity of the INO80-C chromatin remodeler to AP site lesions and UV-induced CPD lesions.

**Figure 3:**
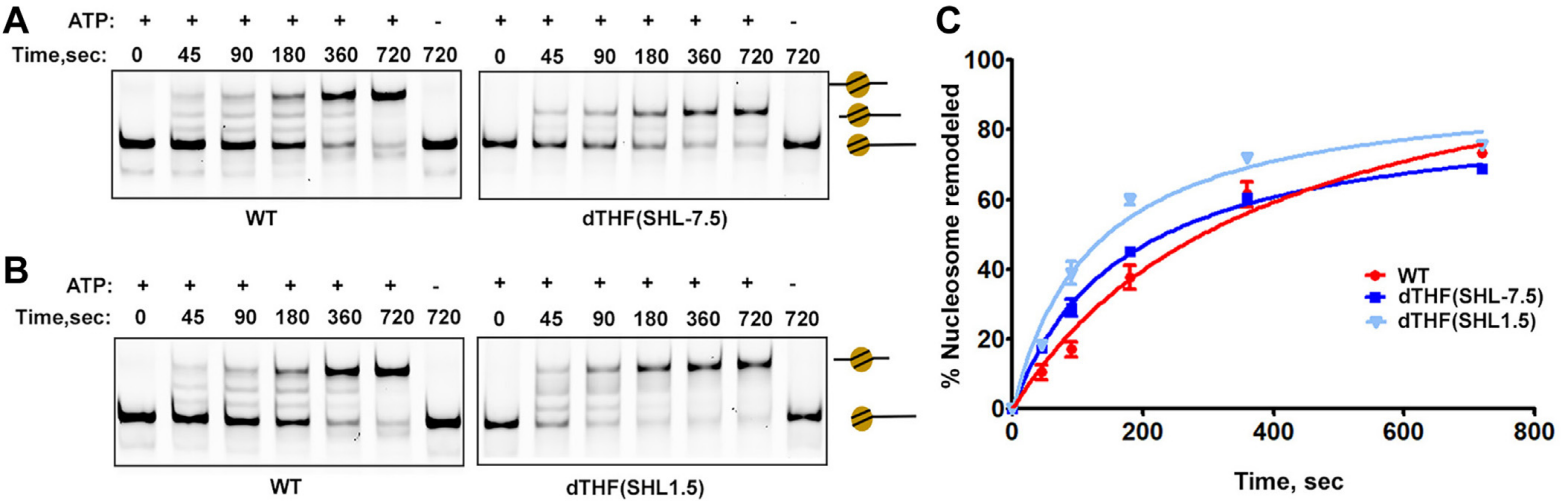
**Nucleosome sliding with DNA lesions outside INO80-C-binding sites.***A*, representative Native-PAGE showing sliding on canonical nucleosomes (WT) and nucleosomes with double THF at SHL-7.5 position. The position of the different nucleosome species on the gel was denoted by schematics on the right, including the band representing the remodeled end-positioned nucleosome where DNA translocation was stalled at SHL-7 site. *B*, representative Native-PAGE shows sliding on WT nucleosome and nucleosomes with double THF at SHL1.5 site. *C*, quantification of (*A* and *B*). Data are mean ± SD, n = 3.

We next extended our analysis to comprehend the interactions between INO80-C and damaged nucleosomes beyond the immediate binding sites. Lesions were introduced at two additional positions (SHL-7.5 and SHL1.5) on the nucleosome (Fig. 2*A*). We hypothesized that SHL1.5 lesions would have minimal impact on DNA translocation, as this region lacks interaction with the Ino80 motor and the Arp5-Ies6 module during sliding. Conversely, SHL-7.5 lesions contact the Ino80 subunit when the extra DNA shifts toward the nucleosome center. The outcome revealed uninterrupted nucleosome sliding on dTHF(SHL1.5) nucleosomes, similar to wild-type nucleosomes (Fig. 3, *B* and *C*). However, the INO80-C complex demonstrated only partial remodeling with dTHF(SHL-7.5) nucleosomes (Fig. 3, *A* and *C*). In essence, these findings not only validate our hypothesis but also corroborate our previous observations regarding the extreme sensitivity of INO80-C complexes to DNA lesions. Moreover, they suggest that the direct interactions between INO80-C and nucleosomes are pivotal not only for coupling ATP hydrolysis to the mechanical force enabling DNA translocation but also for detecting and sensing DNA lesions on nucleosomes.

### Nucleosome-stimulated ATP-hydrolysis of the Ino80 motor impaired by DNA damage

**Supporting Figure S2:**
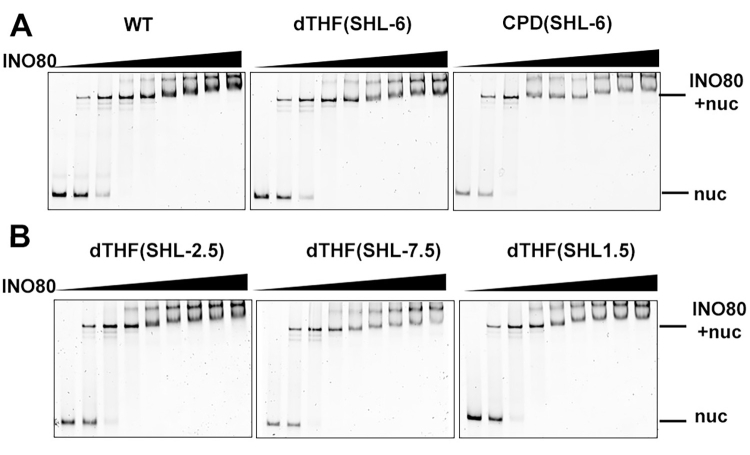
**INO80-C ΔN complex binds to damaged nucleosomes.***A*, 4% Native-PAGE gels showing binding of recombinant INO80-C ΔN complex with canonical (WT) nucleosome (*left*), dTHF(SHL-6) nucleosome (*middle*), and CPD(SHL-6) nucleosome (*right*). 70 nM nucleosomes were mixed with increasing amounts (0, 35, 70, 105, 140, 175, 210, 245, 280 nM) of enzyme in a total volume of 10 μl reaction for each nucleosome. *B*, 4% Native-PAGE gels showing binding of recombinant INO80-C ΔN complex with dTHF(SHL-2.5) nucleosome (*left*), dTHF(SHL-7.5) nucleosome (*middle*), and dTHF(SHL1.5) nucleosome (*right*). 70 nM nucleosomes were mixed with increasing amounts (0, 35, 70, 105, 140, 175, 210, 245, 280 nM) of enzyme in a total volume of 10 μl reaction for each nucleosome.

Our subsequent focus was to assess the influence of DNA lesions on both nucleosome binding and the nucleosome-stimulated ATPase hydrolysis of the INO80-C **Δ**N complex. To achieve this, we employed an EMSA-based binding assay to evaluate the affinity of the INO8-C **Δ**N complex towards nucleosomes containing dTHF at the superhelical locations previously mentioned (SHL-6, SHL-2.5, SHL-7.5, and SHL1.5). We observed complex formation occurring at an equivalent INO80-C **Δ**N to nucleosome ratio across the different damaged nucleosomes tested (Fig. S2, *A* and *B*). These findings indicate that nucleosome lesions have negligible impact on the binding of INO80-C to its substrates.

**Figure 4:**
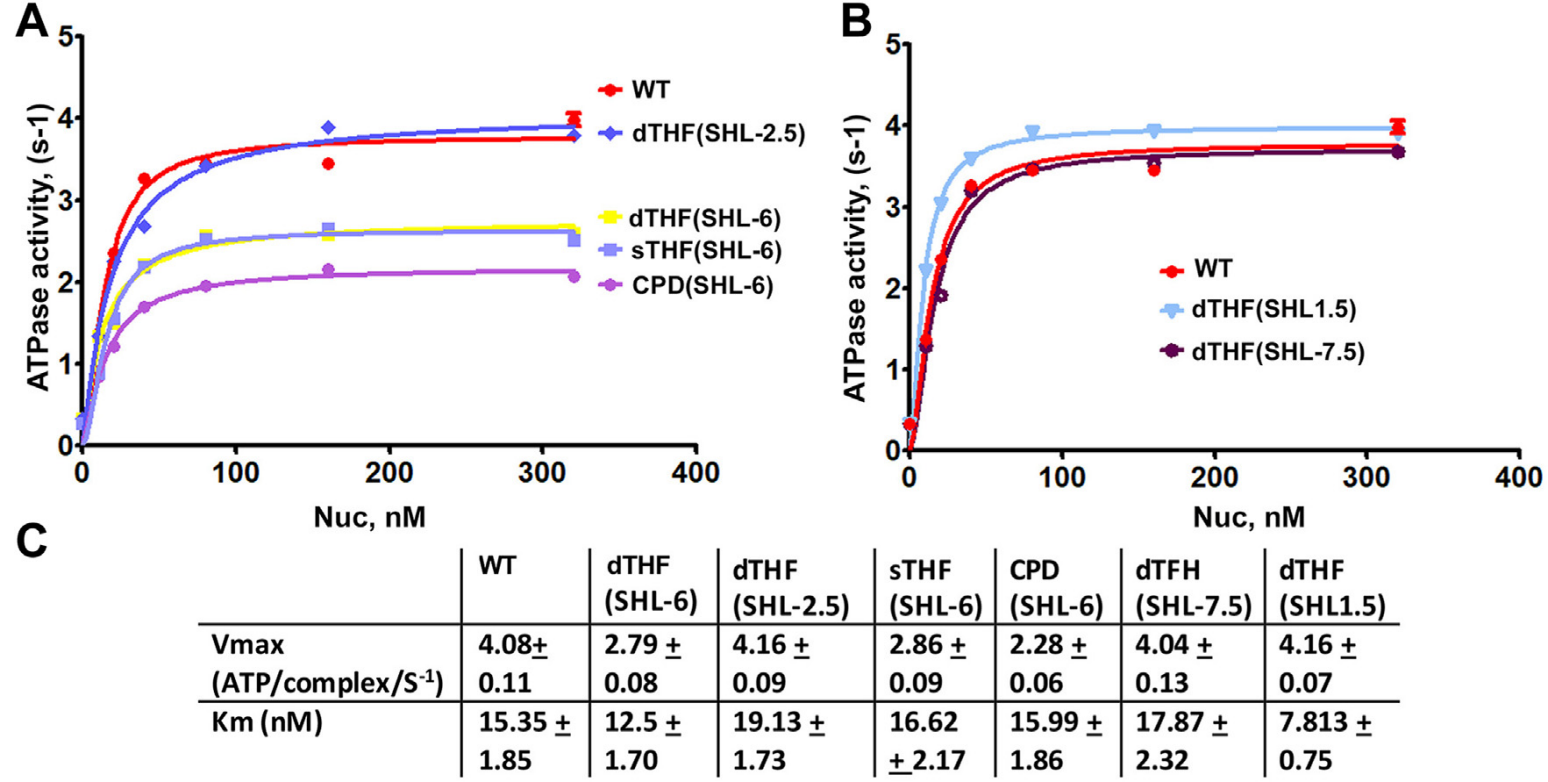
**Nucleosome-stimulated ATPase assay with damaged nucleosomes.***A*, ATPase rate of INO80-C ΔN complex over a range of concentration of nucleosomes. Canonical (WT) nucleosomes and damaged nucleosomes with lesions on SHL-6 and SHL-2.5 were assayed. The ATP and enzyme concentrations in the assay were kept constant at 1 mM and 80 nM, respectively. Data are mean ± SD, n = 3. *B*, the ATPase rate of INO80-C ΔN complex over a range of damaged nucleosome concentrations. Canonical (WT) nucleosomes and damaged nucleosomes with lesions on SHL-7.5 and SHL1.5 were assayed. The ATP and enzyme concentrations in the assay were kept constant at 1 mM and 80 nM, respectively. Data are mean ± SD, n = 3. *C*, ATPase kinetic data for WT and the damaged nucleosomes.

To delve deeper into the inhibitory effects of DNA lesions on INO80-C remodeling activities, we conducted measurements of nucleosome-stimulated ATPase activity of INO80-C **Δ**N through a NADH+-coupled assay. In these reactions, varying nucleosome concentrations were used alongside a saturated ATP concentration of 2 mM. The ATPase hydrolysis rate with canonical undamaged nucleosomes measured 4.08 ± 0.11 (S^−1^) (Fig. 4*C*), aligning with previously reported values (22, 25, 29). Notably, our data unveiled an approximate twofold reduction in the ATP-hydrolysis rate when damaged nucleosomes with lesions at SHL-6 were used. The V_max_ values were nearly two-fold lower with CPD(SHL-6) nucleosomes, and approximately 1.4-fold lower for both dTHF(SHL-6) and sTHF(SHL-6) nucleosomes (Fig. 4, *A* and *C*). These results suggest that lesions at the SHL-6 position significantly compromise ATP hydrolysis of the catalytic subunit Ino80. Conversely, the ATP hydrolysis rate on nucleosomes bearing lesions at other sites (SHL-2.5, SHL-7.5, and SHL1.5) was similar to those observed with canonical nucleosomes (Fig. 4, *A*–*C*). Additionally, the Km values derived from the assay with various lesion-containing nucleosomes were similar, except with the dTHF(SHL1.5) nucleosome (Fig. 4*C*). Given the saturating nucleosome concentrations used in our experiments, Km can be approximated as Kd. These findings further underscore that DNA lesions do not significantly hinder INO80-C **Δ**N binding to the nucleosome, aligning with the results from our EMSA-based nucleosome-binding experiments. Consequently, we deduce that DNA lesions compromise the nucleosome-stimulated ATP-hydrolysis by the Ino80 motor, which subsequently contributes to the impaired DNA translocation of the enzyme on damaged nucleosomes.

### INO80-C stimulates APE1 endonuclease activity

APE1 is a critical enzyme to initiate the removal of the AP sites on DNA through BER. Its action generates a single-strand nick on DNA, which is subsequently repaired by nucleotide gap-filling and sealing in the DNA backbone (30). A previous study demonstrated that the SWI/SNF-like ATPase, Cockayne Syndrome B (CSB) protein, physically interacts with APE1 and stimulates its AP site incision activity on DNA substrates (31). Interestingly, ATP's effects on APE1 endonuclease activity have proven complex (32), with ATP presence inhibiting APE1 incision *in vitro*.

**Figure 5:**
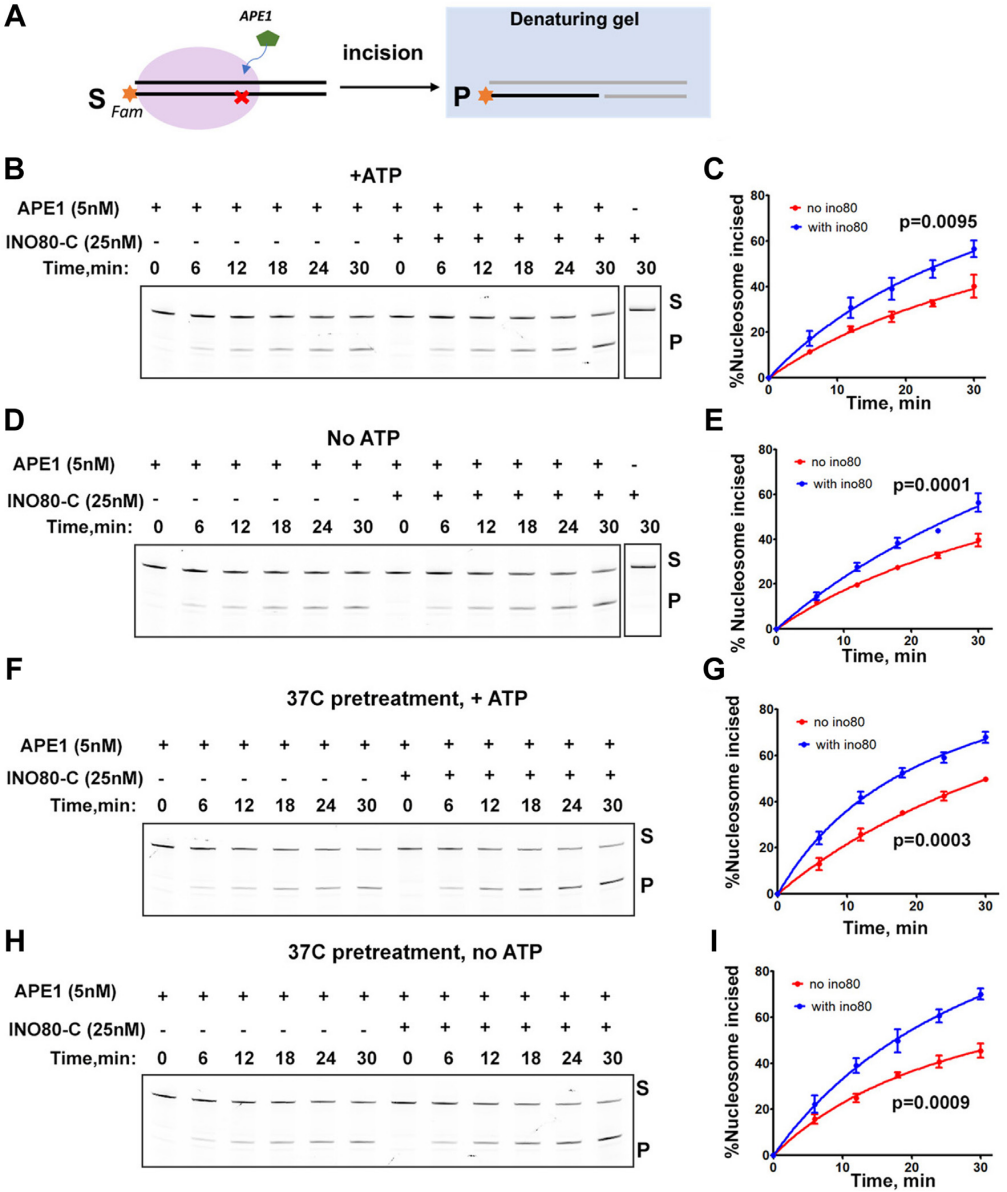
**INO80-C stimulates APE1 endonuclease activity.***A*, schematic showing the APE1 incision assay on the Fam-labeled (*yellow star*) 0N80 nucleosome. A *red cross* denotes the double THF on DNA at SHL-6 site. Substrate (S) and product (P) were separated in a denaturing gel. *B*, denaturing gel showing APE1 incision of nucleosome substrates with ATP present, without INO80-C (lane #1–6) and with INO80-C (lane #7–12). INO80-C alone (last lane) was assayed in the same condition. Each reaction was carried out with 100 nM nucleosome. APE1 and INO80-C concentrations were indicated. *C*, quantification of (*B*) as the percentage of DNA cleaved. Data are mean ± SD, n = 3. *p* value was shown. *D*, denaturing gel showing APE1 incision of the nucleosome substrate without ATP, without INO80-C (lane #1–6) and with INO80-C (lane #7–12). 100 nM nucleosome were used in each reaction. INO80-C alone (last lane) was also assayed in the same condition. *E*, quantification of (*D*) as the percentage of nucleosome DNA cleaved. Data are mean ± SD, n = 3. *p* value was indicated. *F*, denaturing gel showing APE1 incision reaction after APE1-INO80-C pre-treatment at 37 °C with ATP. 100 nM nucleosome were used in each reaction. *G*, quantification of (*F*). Data are mean ± SD, n = 3. *p* value was shown. *H*, denaturing gel showing APE1 incision reaction after APE1-INO80-C pre-treatment at 37 °C without ATP. 100 nM nucleosome were used in each reaction. *I*, quantification of (*H*). Data are mean ± SD, n = 3. *p* value was shown.

A recent quantitative mass spectrometry study identified Ino80 enrichment at AP sites in *S. cerevisiae* while investigating the interactors involved in recognizing and repairing common DNA lesions (33). This observation suggests a potential role of INO80-C in the BER pathway. To delve deeper, we explored the interplay between endogenous INO80-C and human APE1. Using the dTHF(SHL-6) nucleosome substrate with a 6-Fam-label at the DNA 3′ end, we evaluated the impact of INO80-C on APE1 endonuclease activity through a reconstituted incision assay (Fig. 5*A*). As depicted in Figure 5, *B* and *C*, APE1 cleaved approximately 40% of the dTHF(SHL-6) nucleosomes after 30 min. The presence of INO80-C led to notably increased APE1 incision efficiency, with about 56% of the dTHF(SHL-6) nucleosomes cleaved by the end of the reaction (Fig. 5, *B* and *C*). Notably, our data indicate that ATP is not required for the INO80-C-facilitated APE1 incision, as a comparable increase in incision efficiency was observed in the absence of ATP (Fig. 5, *D* and *E*). In both conditions (with and without ATP), we confirmed that INO80-C alone did not affect AP site incision on dTHF(SHL-6) nucleosomes.

We next explored the potential influence of INO80-C on the conformational stability of APE1. Previous studies have documented conformational changes of APE1 triggered by metal ions, redox inhibitors, and substrate binding (32, 34, 35). These conformational shifts are believed to mediate APE1's redox and nuclease activities. In addition, abasic DNA binding has been shown to induce significant thermal stabilization and conformational change in APE1 (34). We thus hypothesize that interactions between INO80-C and APE1 might similarly affect APE1's conformation and stability. We subjected the APE1 alone sample and the APE1-INO80-C mixture to 37 °C pre-treatment to enhance their structural flexibility and protein instability. The pre-treated enzyme/enzyme mixture was then used in the incision reactions conducted at room temperature. Our results demonstrated that APE1's endonuclease activity remained consistent regardless of 37 °C pre-treatment, while the proportion of incised nucleosomes rose to 70% in reactions containing the pre-treated APE1-INO80-C enzyme mixture (Fig. 5, *F* and *G*). Similar results were observed when ATP was absent in the reactions (Fig. 5, *H* and *I*). These results imply that INO80 may enhance APE1 thermal stability, thereby boosting its endonuclease activity. In summary, these findings highlight the ability of INO80-C to enhance APE1 endonuclease activity on the nucleosomal template *in vitro*. Importantly, this novel property of INO80-C does not hinge on ATP hydrolysis.

## Discussion

The conventional understanding of remodeling factor action in DNA damage repair is that they ensure accessibility of DNA at damaged sites. However, different remodelers exhibit varying sensitivities to DNA defects, influencing their ATP-dependent chromatin remodeling activities. For instance, SWI/SNF, mostly unaffected by DNA lesions, promotes the excision of AAF-G adduct by enhancing DNA accessibility on AAF-G nucleosomes (5). Another study revealed that DNA mismatches and single nucleotide insertions disrupt nucleosome sliding by the CHD1 remodeling factor (36). While nucleosome sliding by CHD1 was entirely hindered on nucleosomes containing AP sites on both DNA strands, a single AP site on one strand only decelerated DNA translocation by CHD1 (36).

On the opposite end of the spectrum, our current study demonstrated that the INO80-C chromatin remodeler is highly sensitive to both AP sites and CPD lesions. A single AP site is sufficient to halt DNA translocation by INO80-C. Given the conserved nature of INO80 cores in both yeast and humans, this property likely applies to the human INO80 complex. As INO80 is crucial for repairing various DNA lesions, our findings suggest an unconventional remodeling mechanism for INO80 in damage repair. The precise nature of this mechanism remains an open question. One possibility is that INO80 acts as a lesion detector in the initial steps of damage repair, orchestrating downstream repair factors to facilitate efficient repair processes. Our data indicate that the essential subunits of INO80-C, the catalytic Ino80 motor, and the Arp5-Ies6 module, sense DNA lesions to prevent DNA translocation on damaged nucleosomes. This might be a fundamental property of ATP-dependent chromatin remodelers *in vivo*, where they utilize their lesion-sensitive remodeling activity to preserve genome stability and prevent cryptic transcription on damaged chromatin. Supporting this notion, a recent study showed that INO80 recruits multiple ubiquitin-conjugating factors to DNA damage sites, which modulates the composition and compaction of local chromatin by facilitating histone degradation (37). This study and our findings suggest that the INO80 chromatin remodeler's capacity to induce structural changes and enhance chromatin accessibility at damaged sites primarily relies on its ability to recruit downstream repair factors, rather than on its ATP-dependent DNA translocation activity.

Furthermore, we have observed that INO80-C enhances the endonuclease activity of human APE1 on damaged nucleosomes, suggesting a plausible functional interaction between INO80 and this vital BER enzyme. As our experiments involved yeast INO80-C, human APE1, and *Xenopus* nucleosomes, these results and speculations must be confirmed through future experiments using components from the same species (yeast or human). Nevertheless, given the high conservation of the INO80 complex across various eukaryotic species, our observation is likely a common phenomenon involving the INO80 complex. Interactions between remodelers and APE1 have been previously reported, as seen with another SWI/SNF-related chromatin remodeler, CSB, which was shown to facilitate APE1 incision on DNA substrates (32, 34). These findings suggest that functional interactions with this BER enzyme might be more common within the remodeler superfamily than previously understood. Due to the known conformational dynamics of APE1 and its functional implications, we propose that INO80 forms a transient complex with APE1, which may preferentially bind and stabilize the active and lesion-binding-competent form of the endonuclease. Alternatively, INO80 may promote the turnover of APE1 on damaged nucleosomes to enhance BER repair, similar to a mechanism recently proposed for the UV-damaged DNA-binding (UV-DDB) complex (38). Further *in vitro* studies are required to dissect the intricacies of INO80-APE1 interactions and elucidate the underlying mechanism of INO80-stimulated APE1 incision.

## Experimental procedures

### Expression and purification of recombinant INO80-C complexes

Genes for all subunits of the INO80-C complex were cloned from the *S. cerevisiae* genomic DNA. Genes were then cloned into pACEBAC1 vector using the MultiBac system (Geneva Biotech) to produce four bacmids (Ino80/Arp5/Ies6/Ies2, Rvb1/Rvb2, Arp4/Arp8/Act1/Ies4/Taf14, and Nhp10/Ies1/Ies3/Ies5). Baculoviruses were produced using Sf9 insect cells (Invitrogen). High Five cells (Invitrogen) were co-infected with the corresponding bacmids for protein production. All four bacmids were used to produce the INO80-C full complex, where the Ino80 subunit has a C-terminal twin-strep tag. To produce the ten-subunit INO80-C ΔN sub-complex, a truncated form of the Ino80 subunit (residue 470 from 1490) with a C-terminal Twin-Strep tag was used. The INO80-C ΔN sub-complex contains Ino80ΔN, Arp5, Ies6, Ies2, Rvb1, Rvb2, Arp4, Arp8, Act1, and Ies4. Cells were cultured for 60 h at 27 °C post-infection before harvesting.

Cell pellets (from 1 L of culture) were suspended in buffer A [50 mM Tris-HCl (pH 7.5), 400 mM NaCl, 10% glycerol, 2 mM MgCl2, 1 mM TCEP supplemented with protease inhibitor cocktail (Roche) and benzamidine hydrochloride] followed by sonication. The cell lysate was cleared by ultracentrifugation at 44,000*g* at 4 °C for 1 h and filtered through a 0.45 μm filter before loading onto a Strep-XT column (Cytiva). The column was washed in Buffer B [50 mM Tris-HCl (pH 7.5), 250 mM NaCl, 10% glycerol, 2 mM MgCl2, 1 mM TCEP]. The Strep Tactin-bound protein was then eluted with buffer C [50 mM biotin, 50 mM Tris-HCl (pH 7.5), 150 mM NaCl, 10% glycerol, 2 mM MgCl2, and 1 mM TCEP]. Eluted fractions were then applied to an HQ column (Poros) and further purified using a salt gradient (200 mM NaCl to 1 M NaCl). Fractions were analyzed by SDS-PAGE, and the fractions containing the pure complex were pooled, concentrated (to 2 μM), and flash-frozen in liquid nitrogen for storage.

### Purification of the endogenous yeast INO80-C complex

Tandem affinity purification (TAP) was performed to purify the endogenous INO80-C complex from an *S. cerevisiae* strain encoding TAP-tagged Ino80 (Open Biosystems). Yeast culture was grown in YPD media. Harvested cells were washed once with water. The cells were lysed in buffer E (20 mM HEPES, pH 7.5, 350 mM NaCl, 10 % glycerol, 0.1 % Tween, and 0.5 mM DTT) supplemented with protease inhibitors using a ball mill in the presence of liquid nitrogen. Lysate was clarified at 40,000*g* at 4 °C for 1 h. Cleared lysate was incubated with IgG-Sepharose (GE Healthcare) at 4 °C for 2 h, and eluted by TEV protease (Invitrogen) cleavage at 4 °C overnight. The TEV elute was then incubated with Calmodulin-Sepharose beads (Agilent Technology) in buffer E plus 2 mM CaCl_2_ at 4 °C for 2 h, followed by elution in buffer E plus 10 mM EGTA. Purified proteins were concentrated with VIVASPIN concentrators (Sartorius) and dialyzed against buffer E with 1 mM DTT. Subunit composition was confirmed by SDS-PAGE and mass spectrometry.

### Histone purification and octamer reconstitution

*Xenopus laevis* histones H2A, H2B, H3, and H4 were expressed in bacteria, purified, and used for nucleosome reconstitution as previously described (39). Specifically, histone H2A and H2B were expressed in BL21 (DE3) pLysE cells. H3 and H4 were expressed in BL21 (DE3) cells. An equal molar of each histone was mixed and incubated for 2 h in unfolding buffer [7 M guanidine HCl, 20 mM Tris, pH 7.5, and 10 mM DTT] followed by dialysis against at least three changes of refolding buffer [10 mM Tris (pH 7.5), 1 mM EDTA, 2 M NaCl and 1 mM DTT] at 4 °C. Octamer was concentrated and purified by gel filtration chromatography using a Superdex200 increase 10/300 Gl column (GE healthcare).

### Damaged DNA

End-positioned 0N80 (80 base pairs of extranucleosomal DNA at one entry/exit site) Widom DNA (WT) was amplified by PCR using primers (0N80-F 5′-CTGGAGAATCCCGGTGCCGAG-3′ and 0N80-R 5′-TCGGTACCCGGGGATCCTCTA-3′) and from the plasmid pGEM-3z/601 (40), a kind gift from Jonathan Widom (Addgene plasmid #26656) (40). 0N80 Widom DNAs containing lesions sTHF, dTHF, or CPD were generated using the ligation strategy as described (41). Briefly, a set of oligonucleotides were synthesized for each damaged Widom 601 sequence. Sequence information of these oligoes is included in the Table S1. Oligoes in each set were annealed and then treated with ligase. Oligonucleotides were mixed in equimolar amounts in an annealing buffer [10 mM Tris-HCl pH7.5, 50 mM NaCl, 1 mM EDTA] to a final concentration of 50 uM of each oligo. Annealing was done by heating the reaction to 95 °C for 5 min and cooling it to room temperature at 1 °C per minute. T4 ligase buffer and Salt-T4 DNA Ligase (NEB) were added to the annealing reaction at 0.125x of the annealing reaction volume. Ligase reactions were kept at room temperature for 24 h and then stored at 4 °C until purification. The DNA fragment was purified essentially as described (42). Briefly, a MonoQ 4.6/10 PE (Cytiva) column with a long gradient (70 Column Volume, from 0.6 M NaCl to 0.8 M NaCl, flow rate of 0.1 ml/min) was used to separate the DNA fragments. Peak fractions were combined followed by DNA precipitation through Isopropanol. DNA pellets were dissolved in TE buffer (10 mM Tris-HCl pH7.5, 0.1 mM EDTA) to a final concentration of 0.5 to 1 mg/ml. The DNAs were then resolved on 15% TBE-Urea gels (Invitrogen) and stained with SYBR-Gold for visualization, to ensure the purity and that negligible nicks and gaps are present in the DNA templates.

### Nucleosome reconstitutions

To reconstitute damaged nucleosomes, the octamer was mixed with the 0N80 601 Widom DNA containing the specific DNA lesion in high-salt buffer [10 mM Tris, pH 8.0, 2 mM EDTA, 2 M NaCl and 2 mM 2-Mercaptoethanol (βME)]. The mixture was then dialyzed overnight into low salt buffer [10 mM Tris, pH 8.0, 2 mM EDTA, 5 mM NaCl, and 2 mM βME] as described (39). The optimal ratio of DNA to octamer was determined empirically through careful titrations and examination by the electromobility shift assay (EMSA). Approximately equal molars of octamer and DNA were used.

### Nucleosome sliding assay

Unless otherwise stated, 0N80 end-positioned nucleosomes with and without DNA lesions (140 nM) were incubated with INO80-C complexes (50 nM) in sliding buffer [25 mM HEPES pH8.0, 50 mM NaCl, 5% glycerol, 1 mM TCEP and 2 mM MgCl_2_] in a final volume of 10 μl at room temperature. For reactions presented in Figure 1, different concentrations of NaCl were used. Sliding was initiated by adding 1 mM ATP, and the reaction was quenched by adding 5 mM EDTA and 0.2 mg/ml lambda DNA (NEB). Reactions at different time points were collected and resolved on 6% Native-PAGE gels at 4 °C (100 V, 90 min, 1× TBE). Gels were stained with SYBR-GOLD (GoldBio) before imaging on a Typhoon imager (Cytiva). Quantification of the gels was done using ImageJ software version 1.53e. The percentage of fully remodeled nucleosomes was plotted against time using GraphPad (Prism) software.

### NADH-coupled ATPase assay

NADH–coupled absorbance ATPase assays (43) were used to determine the rate of nucleosome-stimulated ATP hydrolysis of the INO80-C ΔN sub-complex. A range of nucleosome concentration (0, 10, 20, 40, 80, 160, 320 nM) was assayed. For each reaction, nucleosome with specific lesions on various positions were mixed with 80 nM complex in a final volume of 70 μl in assay buffer (25 mM HEPES pH 8, 50 mM NaCl, 1 mM TCEP, 2 mM MgCl2, 5% glycerol) with 0.5 mM phosphoenolpyruvate, 1 mM ATP, 0.1 mM NADH, and lactate dehydrogenase and pyruvate kinase (25 U/ml, Sigma-Aldrich). Reactions were conducted at 30 °C by mixing all components immediately before transferring to nonbinding, white, 96-well plates (Greiner Bio-One). The change in absorbance at 340 nm was monitored with a BioTek Synergy Neo2 multi-mode reader (Agilent) for 1 h. The ATPase rate was determined using maximal initial linear rates. Reaction kinetics were derived assuming a Michaelis-Menten model. Reactions for each substrate were done in triplicates.

### APE1 incision assay

APE1 incision reactions were carried out in a reaction mixture (30 μl) containing incision buffer [25 mM HEPES pH8.0, 75 mM NaCl, 2.5% glycerol, 1 mM TCEP, and 2 mM MgCl_2_, and 0.1 mg/ml bovine serum albumin (BSA)]. 100 nM nucleosomes (containing double THF at SHL-6 position and 6-FAM label on 3′ of the 601 DNA on the same strand) and 5 nM human APE1 endonuclease (NEB, cat # M0282S) were included in each reaction, with or without 25 nM endogenous INO80-C complex, with or without 1 mM ATP. APE1 complex was excluded in the control experiments. Reactions were incubated at room temperature, and samples were collected at a series of time points up to 30 min. The reaction was quenched by adding an equal volume of 2X Urea-TBE loading buffer (Invitrogen). The samples were then incubated at 95 °C for 5 min to separate the double-strand duplex and resolved on a 15% Urea-TBE denaturing gel (Invitrogen) to detect products of single-strand DNA. Gels were imaged on a Typhoon imager (Cytiva). The substrate and product bands were quantified using ImageJ software. The percentage of nucleosomes cleaved was plotted against time using GraphPad (Prism) software. The two-way ANOVA test was used to determine whether the differences between data sets are statistically significant using the *p* ≤ 0.05 criterion. Two-way ANOVA test and graphical representation were done using Prims 5 software.

For the 37 °C APE1 incision experiment, APE1 alone or APE1 pre-mixed with endogenous INO80-C complex were incubated at 37 °C for 20 min. The enzyme mixture was then added to the incision buffer containing 100 nM nucleosomes to initiate the reaction (final volume 30 μl). The concentrations of APE1 and INO80 in each reaction were 5 nM and 25 nM, respectively. The reaction was carried out with and without ATP at room temperature, as described above.

### Electrophoretic mobility shift assay

To monitor the binding of the enzyme to nucleosomes, 70 nM damaged nucleosomes were incubated with an increased amount of INO80-C ΔN sub-complex in a final volume of 10 μl in the binding buffer [25 mM HEPES pH7.5, 60 mM NaCl, 5% glycerol, 1 mM TCEP]. The reactions were incubated for 10 min at 30 °C before being resolved on 4% Native-PAGE at 4 °C (100 V, 90 min, 1× TBE). The gels were stained with SYBR-GOLD (GoldBio) and imaged by a Typhoon imager (Cytiva).

### Quantification and statistical analysis

In Figures 2*E*, 3*C*, and 5, *C*, *E*, *G*, and *I*, the average values of three biological replicas were shown with the standard deviation (SD). In Figure 4, *A* and *B*, the average values of three technical replicas were shown with SD. In all cases, reproducible results were obtained. Two-way ANOVA test was used to determine statistically significant differences.

## Data availability

All biochemical data presented in this study including gel images, NADH-coupled assay results, and analysis are available to share upon request. Please contact the corresponding author about data availability.

## Supporting information

This article contains supporting information.

## Conflict of interest

The authors declare that they have no conflicts of interest with the contents of this article.
