# Supplementary material for: DNA-translocation-independent role of INO80 remodeler in DNA damage repairs
Source: J Biol Chem. 2023 Sep 9;299(10):105245. doi: 10.1016/j.jbc.2023.105245 (PMC10570696; doi:10.1016/j.jbc.2023.105245)
Supplement: Supporting Table S1 [file mmc1.docx]

**Supplemental Table 1. List of oligonucleotides used in this study**

F – Tetrahydrofuran (THF)

PP - Cyclobutane pyrimidine dimer

6FAM-Fluorescein

PHOS - phosphorylation

| 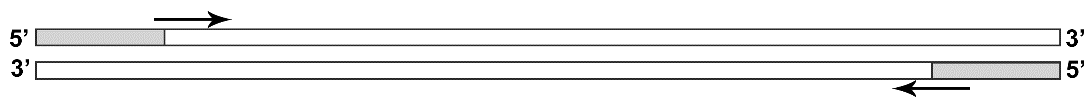  **0N80_Wt** | |
| --- | --- |
| Oligo | Sequence |
| 0N80-F | 5’-CTGGAGAATCCCGGTGCCGAG-3’ |
| 0N80-R | 5’-TCGGTACCCGGGGATCCTCTA-3’ |
| 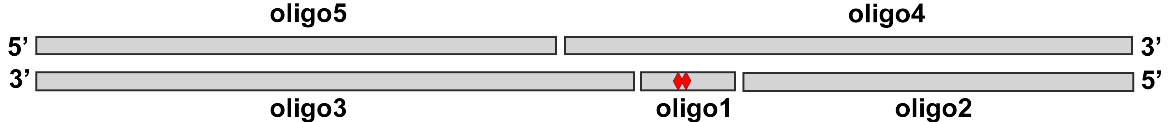  **dTHF(SHL-6)** | |
| Oligo | Sequence |
| dTHF(SHL-6)_1 | 5’-PHOS-CAGGATGTAT**FF**ATCTGACAC-3’ |
| dTHF(SHL-6)_2 | 5’-TCGGTACCCGGGGATCCTCTAGAGTGGGAGCTCGGAACA  CTATCCGACTGGCACCGGCAAGGTCGCTGTTCAATACATGCA-3’ |
| dTHF(SHL-6)_3 | 5’-PHOS-GTGCCTGGAGACTAGGGAGTAATCCCCTTGGCGGTTAAAACGCGGGGGACAGCGCGTACG  TGCGTTTAAGCGGTGCTAGAGCTTGCTACGACCAATTGAGCGGCCTCGGCACCGGGATTCTCCAG-3’ |
| dTHF(SHL-6)_4 | 5’-PHOS-CTCCCTAGTCTCCAGGCACGTGTCAGATATATACATCCTGTGCATGTATTGAACAGCGAC  CTTGCCGGTGCCAGTCGGATAGTGTTCCGAGCTCCCACTCTAGAGGATCCCCGGGTACCGA-3’ |
| dTHF(SHL-6)_5 | 5’-CTGGAGAATCCCGGTGCCGAGGCCGCTCAATTGGTCGTAGCAAGCTCTAGCA  CCGCTTAAACGCACGTACGCGCTGTCCCCCGCGTTTTAACCGCCAAGGGGATTA-3’ |
|  |  |
| 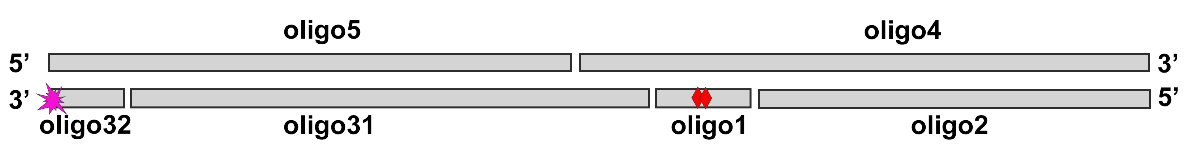  **dTHF(SHL-6)_3’-FAM** | |
| Oligo | Sequence |
| dTHF(SHL-6)_1 | 5’-PHOS-CAGGATGTATFFATCTGACAC-3’ |
| dTHF(SHL-6)_2 | 5’-TCGGTACCCGGGGATCCTCTAGAGTGGGAGCTCGGAACA  CTATCCGACTGGCACCGGCAAGGTCGCTGTTCAATACATGCA-3’ |
| dTHF(SHL-6)_31 | 5’-PHOS-GTGCCTGGAGACTAGGGAGTAATCCCCTTGGCGGTTAAAACGCGGGGGACAGCGCGTA  CGTGCGTTTAAGCGGTGCTAGAGCTTGCTACGACCAATTGAGCGGCCTC-3’ |
| dTHF(SHL-6)_32 | 5’-PHOS-GGCACCGGGATTCTCCAG-6FAM-3’ |
|  |  |
| dTHF(SHL-6)_4 | 5’-PHOS-CTCCCTAGTCTCCAGGCACGTGTCAGATATATACATCCTGTGCATGTATTGAACAGCGACCT  TGCCGGTGCCAGTCGGATAGTGTTCCGAGCTCCCACTCTAGAGGATCCCCGGGTACCGA-3’ |
| dTHF(SHL-6)_5 | 5’-CTGGAGAATCCCGGTGCCGAGGCCGCTCAATTGGTCGTAGCAAGCTCTAGCACCGCTTAAA  CGCACGTACGCGCTGTCCCCCGCGTTTTAACCGCCAAGGGGATTA-3’ |
| 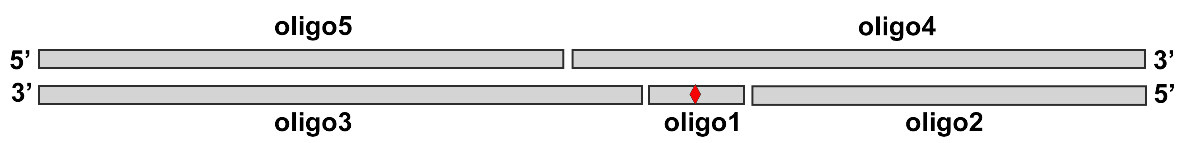  **sTHF(SHL-6)** | |
| Oligo | Sequence |
| sTHF(SHL-6)_1 | 5’-PHOS-CAGGATGTAT**F**TATCTGACAC-3’ |
| sTHF(SHL-6)_2 | 5’-TCGGTACCCGGGGATCCTCTAGAGTGGGAGCTCGGAACA  CTATCCGACTGGCACCGGCAAGGTCGCTGTTCAATACATGCA-3’ |
| sTHF(SHL-6)_3 | 5’-PHOS-GTGCCTGGAGACTAGGGAGTAATCCCCTTGGCGGTTAAAACGCGGGGGACAGCGCGTACG  TGCGTTTAAGCGGTGCTAGAGCTTGCTACGACCAATTGAGCGGCCTCGGCACCGGGATTCTCCAG-3’ |
| sTHF(SHL-6)_4 | 5’-PHOS-CTCCCTAGTCTCCAGGCACGTGTCAGATATATACATCCTGTGCATGTATTGAACAGCGAC  CTTGCCGGTGCCAGTCGGATAGTGTTCCGAGCTCCCACTCTAGAGGATCCCCGGGTACCGA-3’ |
| sTHF(SHL-6)_5 | 5’-CTGGAGAATCCCGGTGCCGAGGCCGCTCAATTGGTCGTAGCAAGCTCTAGCACCGCTTAAAC  GCACGTACGCGCTGTCCCCCGCGTTTTAACCGCCAAGGGGATTA-3’ |
|  |  |
| 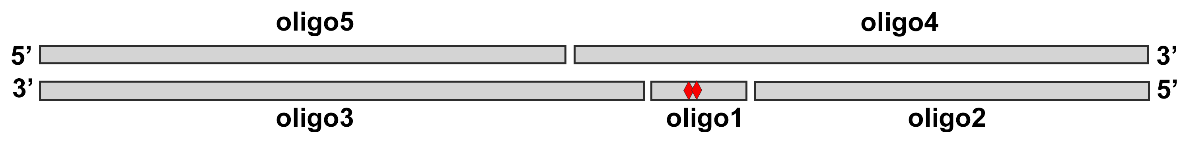  **CPD(SHL-6)** | |
| Oligo | Sequence |
| CPD(SHL-6)_1 | 5’-PHOS-CAGGATGTATPPATCTGACAC-3’ |
| CPD(SHL-6)_2 | 5’-TCGGTACCCGGGGATCCTCTAGAGTGGGAGCTCGGAACA  CTATCCGACTGGCACCGGCAAGGTCGCTGTTCAATACATGCA-3’ |
| CPD(SHL-6)_3 | 5’-PHOS-GTGCCTGGAGACTAGGGAGTAATCCCCTTGGCGGTTAAAAC  GCGGGGGACAGCGCGTACGTGCGTTTAAGCGGTGCTAGAGCTTGCTACG  ACCAATTGAGCGGCCTCGGCACCGGGATTCTCCAG-3’ |
| CPD(SHL-6)_4 | 5’-PHOS-CTCCCTAGTCTCCAGGCACGTGTCAGATATATACATCCT  GTGCATGTATTGAACAGCGACCTTGCCGGTGCCAGTCGGATA  GTGTTCCGAGCTCCCACTCTAGAGGATCCCCGGGTACCGA-3’ |
| CPD(SHL-6)_5 | 5’-CTGGAGAATCCCGGTGCCGAGGCCGCTCAATTGGT  CGTAGCAAGCTCTAGCACCGCTTAAACGCACGTACGCGCTGT  CCCCCGCGTTTTAACCGCCAAGGGGATTA-3’ |
| 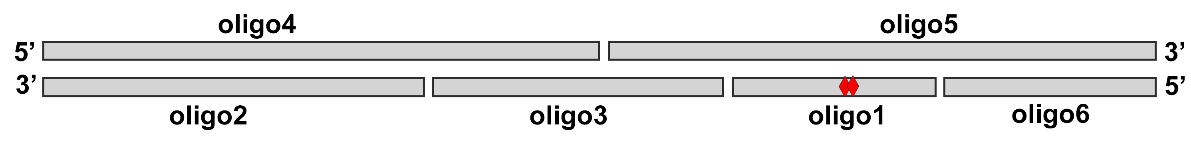  **dTHF(SHL-7.5)** | |
| Oligo | Sequence |
| dTHF(SHL-7.5)_1 | 5’-PHOS-GCACCGGCAAGGTCGCTGTTCAAT**FF**ATGCACAGGATG-3’ |
| dTHF(SHL-7.5)_2 | 5’-PHOS-GGGACAGCGCGTACGTGCGTTTAAGCGGTGCTAGAGCTTGCTACGACCAA  TTGAGCGGCCTCGGCACCGGGATTCTCCAG-3’ |
| dTHF(SHL-7.5)_3 | 5’-PHOS-TATATATCTGACACGTGCCTGGAGACTAGGGAGTAATCCCCTTGGCGGTTAAAACGCGG-3’ |
| dTHF(SHL-7.5)_4 | 5’-CTGGAGAATCCCGGTGCCGAGGCCGCTCAATTGGTCGTAGCAAGCTCTAGCACCGCTT  AAACGCACGTACGCGCTGTCCCCCGCGTTTTAACCGCCAAGGGGATTACTCCCT-3’ |
| dTHF(SHL-7.5)_5 | 5’-PHOS-AGTCTCCAGGCACGTGTCAGATATATACATCCTGTGCATGTATTGAACAGCGACCTTGC  CGGTGCCAGTCGGATAGTGTTCCGAGCTCCCACTCTAGAGGATCCCCGGGTACCGA-3’ |
| dTHF(SHL-7.5)_6 | 5’-TCGGTACCCGGGGATCCTCTAGAGTGGGAGCTCGGAACACTATCCGACTG-3’ |
| 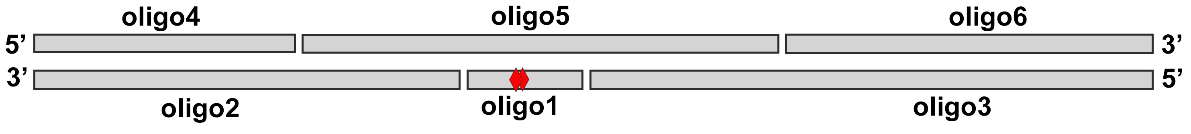  **dTHF(SHL-2.5)** | |
| Oligo | Sequence |
| dTHF(SHL-2.5)_1 | 5’-PHOS-GGAGTAATCCC**FF**TGGCGGTTAAA-3’ |
| dTHF(SHL-2.5)_2 | 5’-PHOS-ACGCGGGGGACAGCGCGTACGTGCGTTTAAGCGGTGCTAGAGCTTGC  TACGACCAATTGAGCGGCCTCGGCACCGGGATTCTCCAG-3’ |
| dTHF(SHL-2.5)_3 | 5’-TCGGTACCCGGGGATCCTCTAGAGTGGGAGCTCGGAACACTATCCGACTGGCAC  CGGCAAGGTCGCTGTTCAATACATGCACAGGATGTATATATCTGACACGTGCCTGGAGACTAG-3’ |
| dTHF(SHL-2.5)_4 | 5’-CTGGAGAATCCCGGTGCCGAGGCCGCTCAATTGGTCGTAGCAAGCTCTAGCAC-3’ |
| dTHF(SHL-2.5)_5 | 5’-PHOS-CGCTTAAACGCACGTACGCGCTGTCCCCCGCGTTTTAACCGCCAAGGGGATTACTC  CCTAGTCTCCAGGCACGTGTCAGATATATACATCCTGT-3’ |
| dTHF(SHL-2.5)_6 | 5’-PHOS-GCATGTATTGAACAGCGACCTTGCCGGTGCCAGTCGGATAGTGTTCCGAGCTC  CCACTCTAGAGGATCCCCGGGTACCGA-3’ |
| 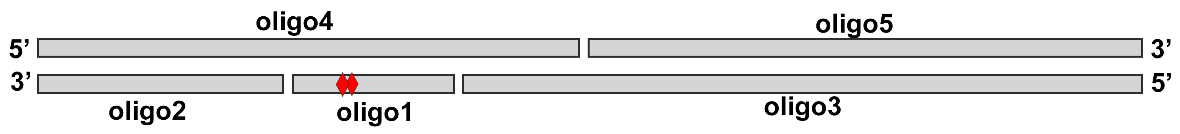  **dTHF(SHL1.5)** | |
| Oligo | Sequence |
| dTHF(SHL1.5)_1 | 5’-PHOS-GGGACAGCGCGTACGTG**FF**TTTAAGCGGT-3’ |
| dTHF(SHL1.5)_2 | 5’-PHOS-GCTAGAGCTTGCTACGACCAATTGAGCGGCCTCGGCACCGGGATTCTCCAG-3’ |
| dTHF(SHL1.5)_3 | 5’-PHOS-ACAGGATGTATATATCTGACACGTGCCTGGAGACTAGGGAGTAATCCCCTTG  GCGGTTAAAACGCGG -3’ |
| dTHF(SHL1.5)_4 | 5’-TCGGTACCCGGGGATCCTCTAGAGTGGGAGCTCGGAACACTATCCGAC  TGGCACCGGCAAGGTCGCTGTTCAATACATGC-3’ |
| dTHF SHL1.5)_5 | 5’-CTGGAGAATCCCGGTGCCGAGGCCGCTCAATTGGTCGTAGCAAGCTCTAGCACCGCTTAAACGCA  CGTACGCGCTGTCCCCCGCGTTTTAACCGCCAAGGGGATTACTCCCT-3’ |
